# The Potential Mechanism of Wuwei Qingzhuo San against Hyperlipidemia Based on TCM Network Pharmacology and Validation Experiments in Hyperlipidemia Hamster

**DOI:** 10.1155/2020/5369025

**Published:** 2020-05-10

**Authors:** Jianliang Li, Chaolu Wang, Lin Song, Shuzhen Cai, Zhiyong Li, Ya Tu

**Affiliations:** ^1^Institute of Chinese Materia Medica, China Academy of Chinese Medical Sciences, Beijing 100700, China; ^2^Medical Research Center, China Academy of Chinese Medical Sciences, Beijing 100700, China; ^3^Wang Jing Hospital of CACMS, China Academy of Chinese Medical Sciences, Beijing 100102, China; ^4^Mongolian Medicine College, Inner Mongolia Medical University, Hohhot 010110, China; ^5^Mongolian Medicine College, Inner Mongolia University of Nationalities, Tongliao 028000, China; ^6^Pharmacy College of Minzu University of China, Beijing 100081, China; ^7^Development Research Center of TCM, China Academy of Chinese Medical Sciences, Beijing, China

## Abstract

Wuwei Qingzuo San (WWQZS), as a renowned traditional Mongolian patent medicine approved by Chinese State Food and Drug Administration, is used to treat hyperlipidemia, indigestion, and other ailments related to disorder of production of essence and phlegm, a typical abnormal metabolism of blood in traditional Mongolian medicine. A combination of network pharmacology and validation experiments in hyperlipidemia hamster is used to understand the potential mechanism of WWQZS for hypolipidemic effects, further for an integrated concept of traditional theory, bioactive constituents, and molecular mechanism for TMM. Through network pharmacology, we obtained 212 components, 219 predicted targets, and 349 known hyperlipidemia-related targets form public database and used Metascape to carry out enrichment analysis of 43 potential and 45 candidate targets to imply numerous BP concerned with metabolism of lipid, regulation of kinases and MF related to lipid binding, phosphatase binding, and receptor ligand activity that are involved in anti-hyperlipidemia. In addition, KEGG pathways that explicated hypolipidemic effect were involved in pathways including metabolism associated with kinase function according to MAPK signaling pathway, AMPK signaling pathway, and PI3K-Akt signaling pathway. Meanwhile, in HFD-induced hamster model, WWQZS could significantly reduce TC and ALT and help decrease TG, LDL-C as well; liver pathological section implied that WWQZS could relieve liver damage and lipid accumulation. Western blot indicated that WWQZS may upregulate CYP7A1 and activate AMPK to suppress the expression of HMGCR in livers. In conclusion, our results suggest that WWQSZS plays important dual hypolipidemic and liver-protective role in livers in HFD-induced hamster model. Through this research, a new reference is also provided to other researches in the study of ethnopharmacology.

## 1. Introduction

Hyperlipidemia is one of the potent risk factors for cardiovascular diseases such as atherosclerosis, myocardial ischemia, and stroke [1, 2]. Excessive dietary fat intake can result in hyperlipidemia, obesity, and nonalcoholic fatty liver disease [3, 4]; meanwhile, the liver is a crucial organ to keep the balance of lipid metabolism, including fat transport, synthesis, and catabolism [5]. Therefore, efficient lipid-lowering by promoting liver function has gained numerous research interests.

Wuwei Qingzuo San (WWQZS) (Tonglaga-5 in Mongolian) is produced by Fuxin Mongolia Medicine Co., Ltd. (Liaoning, China) and approved by the Chinese State Food and Drug Administration (Z21020300). As a traditional Mongolian patent medicine and simple preparation, five herbal medicines in WWQZS are pulverized into powders and mixed; their detailed contents are shown in Table 1.

**Figure 1:**
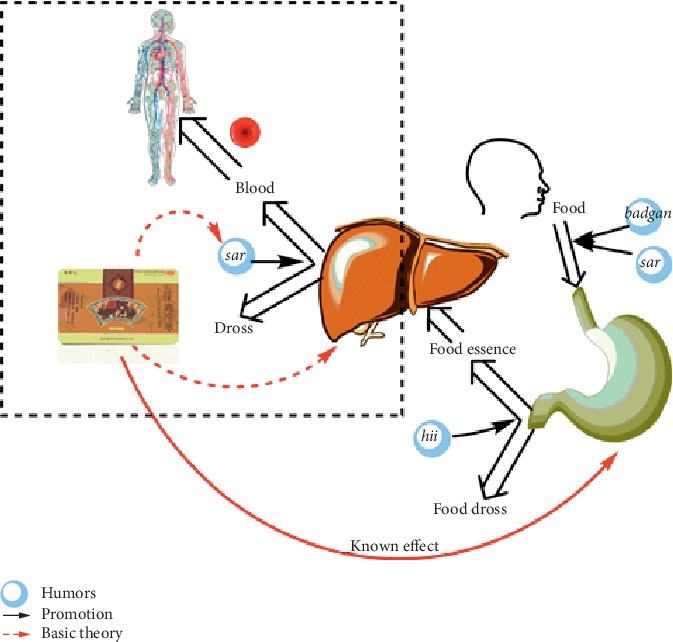
The theories of TMM for WWQZS against hyperlipidemia. Food is digested into the stomach with the aid of badgan and sar; then chyme is divided into food phlegm and food essence by hii in the stomach. *Sar* helps food essence produce essence into the blood to the circulatory system and eliminate phlegm in liver again. WWQZS has a known effect for helping stomach function and a potential effect on liver against hyperlipidemia based on traditional theories of TMM.

WWQZS has applications against poor appetite, indigestion, and stomachache rerecord originated from Pharmacopoeia of The People's Republic of China-2015 Chinese Edition (CH.P); till based on Mongolian medicine theory of “Eliminate Phlegm and Produce Essence” (Qing Zhuo Sheng Hua in Chinese), WWZQS has been used to treat dyslipidemia due to the principles of TMM for helping liver produce essence and eliminate phlegm again (Figure 1). Moreover, it is reported that WWQZS could reduce TC and TG among hyperlipidemia patients [6], and some previous studies have demonstrated that WWQZS can reduce total cholesterol (TG) and triglycerides (TC) in serum and livers in rats with alcoholic fatty liver [7]; meanwhile, WWQZS could inhibit the production of liver *X* receptors (LXRs) and gain expression of peroxisome proliferator-activated receptor *α* (PPAR*α*) in NAFLD rats caused by feeding fatty milk [8]. However, the underlying mechanisms of WWQZS for anti-hyperlipidemia remained unclear and have to be further investigated.

Generally, it is reported there are lots of the researches of traditional medicines in China for figuring how multiple active components of formulas work against illnesses in body found on network pharmacology [9–11]. Therefore, we employed a comprehensive approach that provides an underlying mechanism by combining computational and experimental efforts dependent on numerous targets and pathways based on network pharmacology [12, 13]. Furthermore, we validated experiments in hamsters to further explore the hypolipidemic mechanism of WWQZS based on TCM network pharmacology.

## 2. Methods and Materials

### 2.1. Chemical Components of Wuwei Qingzhuo San

The components and their ADME information of all the five herb medicine in WWQZS were obtained from traditional Chinese Medicine Systems Pharmacology Database and Analysis Platform (TCMSP) (http://lsp.nwu.edu.cn/tcmsp.php) [14] and Chemistry Database [DB/OL] (http://www.organchem.csdb.cn). TCMSP Database and Chemistry Database [DB/OL] both have chemicals of traditional Chinese medicine; moreover, TCMSP database includes targets of components and their pharmacokinetic properties for natural compounds involving oral bioavailability, drug-likeness, intestinal epithelial permeability, blood–brain barrier, aqueous solubility, etc.

### 2.2. ADME Parameters of Candidate Compounds Evaluation

The ADME information of chemicals from TCMSP and Chemistry Database [DB/OL] is found at TCMSP.

We chose the two parameters, namely, oral bioavailability (OB) and drug-likeness (DL), to obtain the potential active ingredients to evaluate the degree of an oral dose of drugs that distribute to the bloodstream and the structural similarity between the compound and the drugs clinically. Ultimately, we defined the screening principle as OB ≥ 30% and DL ≥ 0.1. Others such as ursolic acid and gallic acid from Shiliu, apigenin and caffeic acid from Honghua, D-camphene from Doukou, and (+/−)-isoborneol and alpha-humulene from Bibo as well as Rougui, as active natural products reported before, were omitted based on the principle mentioned before; consequently, we manually added the compounds, which have actual targets from the TCMSP.

### 2.3. Targets Related to Hyperlipidemia Identified in WWQZS

The predicted putative targets of candidate components from five herbs in WWQZS were obtained from TCMSP developed by professor Wang [14] and protein IDs of the targets were converted to gene IDs in UniProt (https://www.uniprot.org/). The interaction for these targets was analyzed by interactive Venngrapher using TBtools v0.5 too. Furthermore, the targets related to hyperlipidemia were collected by the following ways. (1) We searched the keyword with “hyperlipidemia ”and obtained the known targets associated with hyperlipidemia through both TTD（Therapeutic Target Database; http://bidd.nus.edu.sg/BIDD-Databases/TTD/TTD.asp and OMIM database (Online Mendelian Inheritance in Man; http://www.omim.org/). (2) We used the FDA-approved drugs for treating hyperlipidemia in the DrugBank database (https://www.drugbank.ca/) to find hyperlipidemia-related targets. (3) We searched “hyperlipidemia” in the keyword bar to find the targets related to hyperlipidemia by text-mining and downloaded the information of the targets from GeneCards (https://www.genecards.org/). Then we selected the important targets by calculating more than twofold median of the index of “relevance score.” After redundancy was deleted, the known targets related to hyperlipidemia from five databases were collected. Moreover, PPI information was obtained from STRING resource (version 10.5; https://string-db.org/) [15]by limiting organism to “*Homo sapiens*”, setting minimum required interaction score to medium confidence (0.400) as well as max number of interactors to show to 300, which collects and integrates the information about known and predicted protein-protein association data.

### 2.4. Network Construction and Analysis

Three networks of candidate compounds-targets predicted putative targets and known hyperlipidemia-related targets were constructed and visualized by Cytoscape (version 3.6.1) based on the data previously collected. Then, the intersection of networks between predicted putative targets and known hyperlipidemia-related targets network was combined by Merge, a tool of Cytoscape. Afterward, two topological properties of each node, namely, degree and betweenness centrality, in the interaction network was analyzed by NetworkAnalyzer, another tool of Cytoscape, which was the crucial factor to describe the most influential nodes in networks. The greater the value of two topological properties is, the more important the nodes stay in the network.

### 2.5. Gene Ontology and KEGG Pathway Enrichment Analysis

Furthermore, GO annotations and KEGG pathway enrichment analysis for two pairs of targets, namely, potential targets related to hyperlipidemia and candidate targets selected by topological properties in PPI networks, were performed by Metascape (http://metascape.org/gp/), a gene annotation and analysis resource, which can clarify the similarities and differences of two pairs of targets and supply an underlying scientific discovery [16, 17]. First, we chose “Multiple Gene List', then submitted the gene lists of the two targets sets mentioned before, chose the species as ”*Homo sapiens*”, and carried out custom analysis for enrichment analysis of GO annotation and KEGG pathways.

### 2.6. Diets, Wuwei Qingzuo San, and Animals

Normal-fat diets (NFD) and high-fat diets (HFD) containing 10% lard, 10% yolk, 1% cholesterol, and 79% normal-fat diets were prepared by Beijing Keao Xieli Feed Co., Ltd. (Beijing, China); WWQZS (batch number 20170806) was purchased from Fuxin Mongolia Medicine Co., Ltd. (Liaoning, China) and dissolved suspending with 0.5% CMC-Na before use.

Twenty-four male hamsters aged 8 weeks were purchased from Charles River Laboratories in Beijing, China. All experiments were carried out to minimize the suffering of animals. All animal experiments procedures were approved by the Animal Experimental Ethics Review Committee of the Institute of Basic Research for Chinese Medicine, China Academy of Chinese Medical Sciences (license number: SYXK (Beijing) 2016-0021) and all applicable institutional and governmental regulations concerning the ethical use of animals were followed by Beijing Administration Rule of Laboratory Animal. Hamsters were randomly divided into five groups (*n* = 6) after 72 h of adaptation period. Animals were maintained under standard conditions with a 12 h light/dark cycle at a temperature of 23 ± 2°C and humidity of 65–70% and were provided free access to food and water in the Experimental Animal Center, Institute of Basic Theory for Chinese Medicine, China Academy of Chinese Medical Sciences.

### 2.7. Experimental Design

Six hamsters were fed on NFD, and the remaining eighteen hamsters were supplied with HFD. After the five-week induction period, hamsters fed on HFD were randomly divided into three groups due to the serum lipid. Hence, all the hamsters were divided into four groups again: (1) NFD group: NFD + 0.5% CMC-Na; (2) HFD group: HFD + 0.5% CMC-Na; (3) WWQZSL group: HFD + 7.8 g/kg WWQZS; and (4) WWQZSH group: HFD + 3.12 g/kg WWQZS. During the experimental period, body weight was recorded every week. After a 5-week induction period and 8-week supplementation period, the blood was collected via canthus veins after the hamsters were fasted for 12 hours, respectively. After the experimental period, the livers were removed and weighed; meanwhile, the largest lobes of the livers were fixed in 4% paraformaldehyde and the others were stored at −80°C until use.

### 2.8. Determination of TC, TG, LDL-C, HDL-C, AST, and ALT in Serum

The serum was separated by centrifuging at 3000 rpm for 20 min at 4°C and stored at −80°C until analysis. The concentration of serum TC, TG, LDL-C, HDL-C, ALT, and AST was assessed with an automatic biochemical analyzer (OLYMPUS AU480, Japan) following the kit instructions (In Tec PRODUCTS, Inc., Xiamen, China).

### 2.9. Histological Analysis

The largest lobe of the livers was fixed in 4% paraformaldehyde and embedded in paraffin. Sections were obtained and later stained with hematoxylin and eosin (H&E) for the histological examination. Cryosections of liver were stained by 0.2% Oil-Red O and counterstained with hematoxylin to visualize the lipid droplets. Tissue sections were then observed with the microscope (Nikon Eclipse Ci, Japan) and software (Pannoramic Viewer, vision:1.15.3, Hungary).

### 2.10. Western Blot Analysis

Whole-cell protein was prepared from isolated liver and extracted by RIPA buffer. Quantiﬁed protein lysates were separated by SDS-PAGE, transferred to PVDF membrane (Millipore, United States) with 110V at 4°C for 100 min, and incubated overnight at 4°C with primary antibodies, and cholesterol 7*α*-hydroxylase (CYP7A1) (1 : 500, SC-518007, Santa Cruz Biotechnology); phosphoacetyl CoA carboxylase (p-ACC) (1 : 1000, 11818, Cell Signaling Technology); phospho-AMPK*α* (p-AMPK*α*) (1 : 1000, 2535, Cell Signaling Technology); 3-hydroxy-3-methylglutaryl-CoA reductase (HMGCR) (1 : 3000, Ab174830, Abcam); and *β*-actin (1 : 1000, 4967, Cell Signaling Technology) after blocking in TBST containing 5% BSA for 1h at room temperature. Then, membranes were blotted with an appropriate HRP-labeled goat anti-rabbit IgG（*H* + *L*）or anti-mouse IgG (*H* + *L*) (1 : 10000, Jackson) secondary antibody for 1h at room temperature. At last, the membranes were washed with TBST three times again and binding of antibodies was detected using ECL (WBKLS0500, Millipore). Western blot bands were measured using the ImageJ software and normalized using *β*-actin as a control. The results of Western blot analysis were valued by ImageJ software.

### 2.11. Data Analysis

All data were expressed as mean ± SD. Statistical analysis was conducted by one-way ANOVA, followed by LSD *t*-test. The value of *p* < 0.05 was considered statistically signiﬁcant.

## 3. Results

### 3.1. Screening of Candidate Compounds from Five Herbal Medicines in WWQZS

A total of 540 chemicals pieces of information were collected from the two websites and there are 76 from Granati Fructus (Shiliu), 189 from Carthami Flos (Honghua), 104 from Piperis Longi Fructus (Bibo), 71 from Amomi Fructus Rotundus (Doukou), and 100 from Cinnamomi Cortex (Rougui), respectively. The 540 components defined as “collected compounds” were listed in Supplementary Table 1. Further, screening by two principles (OB ≥ 30 %, DL ≥ 0.1) and adding low OB and DL index but highly active activity, we harvested 212 compounds after duplication and removal fitted with the two parameters in the WWQZS, collected as “candidate compounds” in the supplement Table 1. At last, the five herbal medicines, Shiliu, Honghua, Bibo, Doukou, and Rougui, contributed 34, 68, 51, 52, and 55 compounds as “candidate compounds.” Among the 212 candidate compounds, there are no common compounds in the five herbs. However, there are some compounds less than five herbs in common; for example, the only compound that the four herbs, namely Honghua, Bibo, Doukou, and Rougui, have in common is alpha-humulene, a hypolipidemic-activity compound with low OB and DL index.

### 3.2. Construction and Analysis of Compound-Putative Target Network for WWQZS

**Figure 2:**
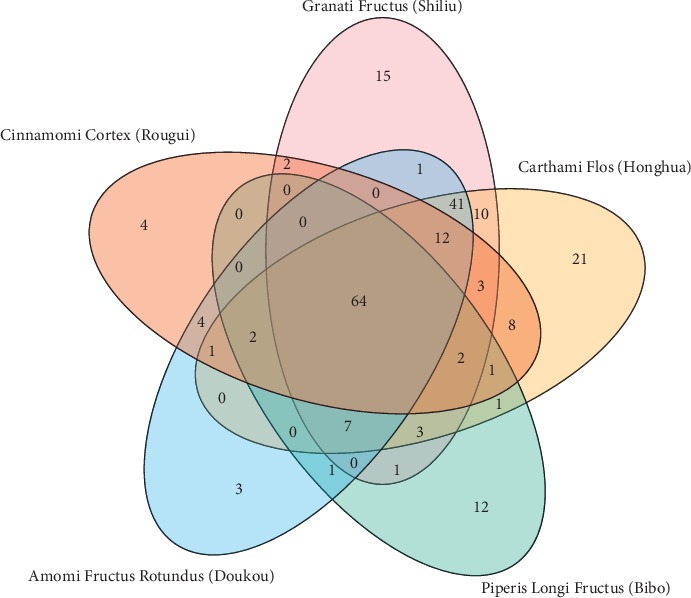
The interaction of targets from five herbs in the Venngrapher. The numbers of the dark red, light red yellow, green, and blue ellipses represent the unique targets of five herbal medicines, respectively, and numbers of overlapped parts represent common targets among the five herbal medicines.

Based on TCMSP, we obtained 219 predicted putative targets for 212 candidate compounds from WWQZS, 161 for Shiliu, 176 for Honghua, 94 for Bibo, 136 for Doukou, and 103 for Rougui (Figure 2).

**Figure 3:**
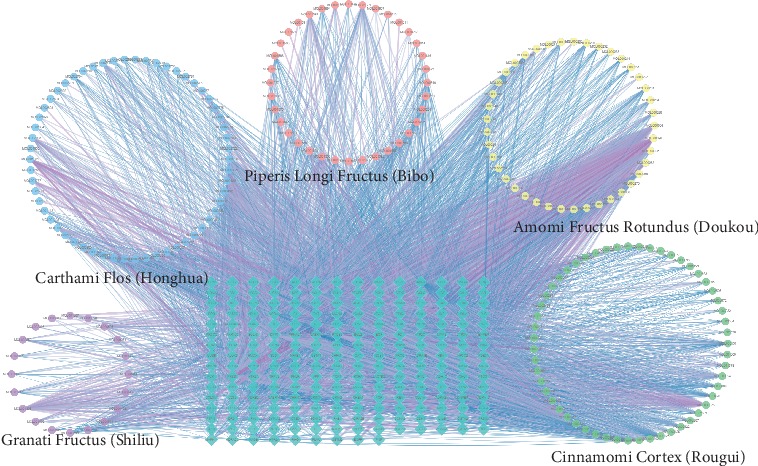
The network of compounds-putative targets for WWQZS. Purple, blue, red, yellow, and green circles represent the components of the Granati Fructus (Shiliu), Carthami Flos (Honghua), Piperis Longi Fructus (Bibo), Amomi Fructus Rotundus (Doukou), and Cinnamomi Cortex (Rougui), respectively. Taking the edge, the larger the value of edge betweenness is, the bigger the size of the edge is; meanwhile, the value is from high to low, color from purple to blue.

Detailed information was described in Supplementary Table 2; moreover, the targets of different herbal medicines from WWQZS overlapped. In other words, different components from the five herbal medicines shared the common and similar targets for the foundation of therapeutic effect. The network of the compound-putative target, consisting of 429 nodes and 4213 edges, was constructed to understand the complex interaction between compounds and their targets (Figure 3).

### 3.3. Identification of Targets for WWQZS against Hyperlipidemia

**Figure 4:**
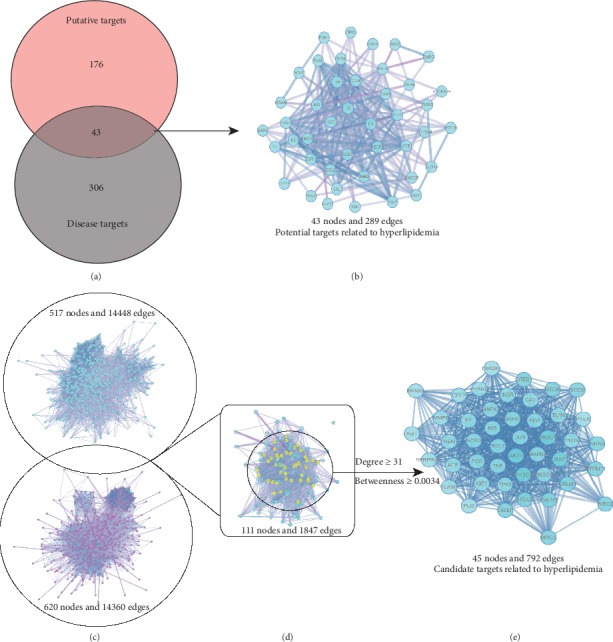
Identification of targets for WWQZS against hyperlipidemia. (a) WWQZS shared 43 targets as potential targets against hyperlipidemia by overlapping it between WWQZS putative targets and disease targets related to hyperlipidemia. (b) The interaction network of 43 overlapped targets. (c) The overlapping between two PPI networks of WWQZS putative targets and disease targets related to hyperlipidemia, consisting of 517 nodes, 14448 edges; 620 nodes, 14360 edges, respectively. (d) The interactive PPI network of putative targets and disease targets, consisting of 111 nodes and 1847 edges. (e) The PPI network of candidate targets related to hyperlipidemia extracted from (d).This network is made of 45 nodes and 792 edges.

Next to 219 predicted putative targets from TCMSP, we gained 349 known targets related to hyperlipidemia based on five existing resources, namely, TTD, OMIM, DrugBank, and GeneCards; the information of those targets was listed in Supplementary Table 3. After linking the overlapped targets between predicted putative targets and known targets related to hyperlipidemia, our analysis showed that the five herbs in WWQZS shared 43 potential targets with known targets related to hyperlipidemia (Figure 4(a)) as a kind of potential therapeutic targets. The PPI network of 43 targets are shown in Figure 4 using STRING database that identified them as potential targets.

The knowledge of protein-protein interaction (PPI) network helps more systemwide understanding of cellular function for the diseases. According to the STRING resource, we constructed a predicted putative targets network (517 nodes and 14448 edges) (Figure 4(c)) and a known hyperlipidemia-related targets network (620 nodes and 14360 edges) (Figure 4(c)). Moreover, the two networks were interested in one merge network consisting of 111 nodes and 1847 edges (Figure 4(d)) to unravel the mechanism of WWQZS against hyperlipidemia. Then, we selected two topological features, degree and betweenness, as important factors in network construction, in order to identify the candidate targets. Subsequently, based on the median values of degree and betweenness being 31 and 0.0034, 45 targets whose network consisted of 45 nodes and 792 edges were identified as candidate targets (Figure 4(e)). Detailed topological feature values and PPI network were shown in Supplementary Table 4.

### 3.4. Enrichment Analysis of Targets for WWQZS against Hyperlipidemia

**Figure 5:**
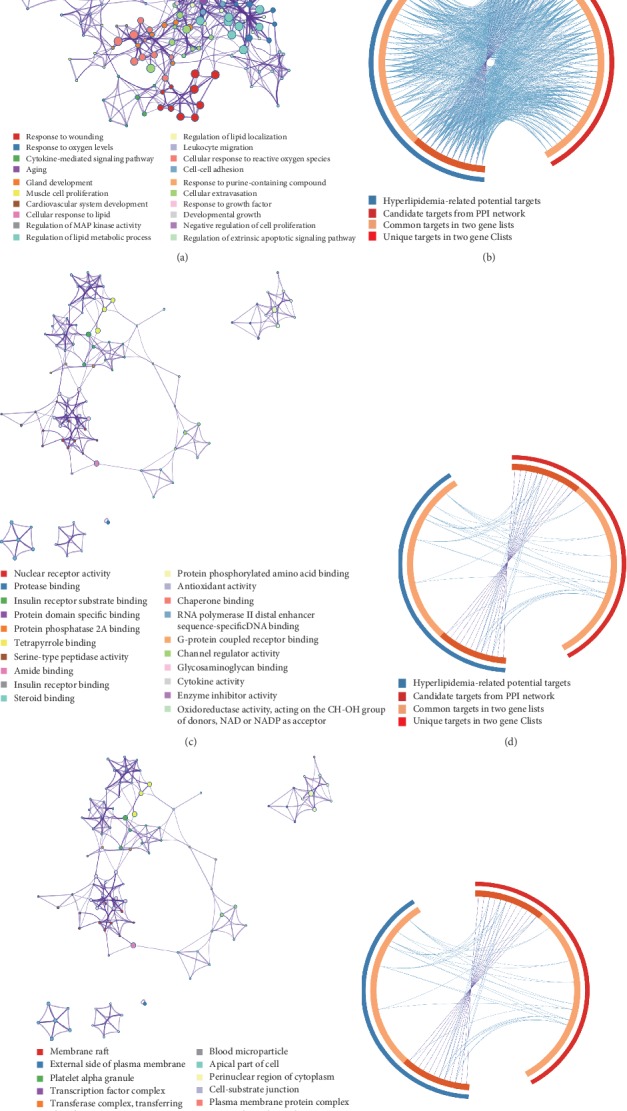
GO enrichment analysis of targets for WWQZS against hyperlipidemia. Enriched GO analysis of term for MF (a), BP (c), and CC (e), colored by ID of the most significant term. Each term is represented by a circle node, where its size is proportional to the number of input genes of the term, and the same color belongs to the same cluster. Terms with a similarity score >0.3 are linked by an edge and the thickness of the edge represents the similarity score. Gene overlap analysis is carried out in two pairs of targets of MF (b), BP (d), and CC (f). Dark orange color represents the genes that appear in two pairs and light orange color represents unique genes in each gene list. Purple lines link the same genes that are shared by two gene lists. Blue lines link represents the different genes where they fall into the same ontology term.

**Figure 6:**
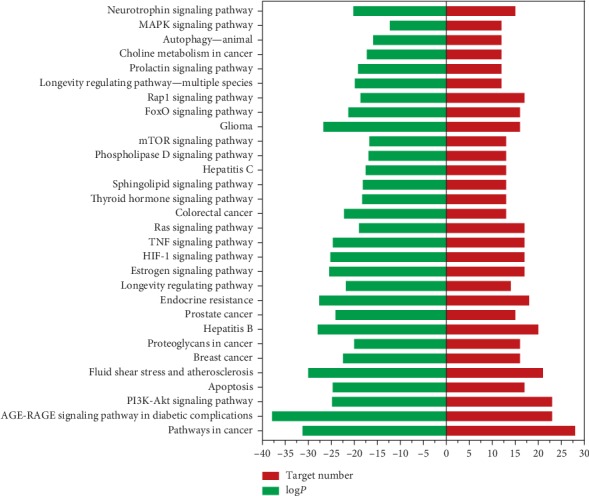
Top 30 significant pathways for WWQZS against hyperlipidemia. The green bar graphs represent log (P) of each term, and red ones represent target numbers in each term.

Based on the previous studies, we first considered both potential targets related to hyperlipidemia and candidate targets selected by topological properties in PPI networks as possible targets for WWQZS against hyperlipidemia. Due to Metascape, terms are grouped into clusters based on their membership similarities; moreover, the most statistically significant *p*-value term in one cluster is chosen to represent the cluster (Figure 5). On the other hand, the Circus plot shows how genes overlap from the two gene lists. The greater the number of purple links and the longer the dark orange arcs implies greater overlap between the two groups of targets and blue links indicate the amount of functional overlap. Moreover, GO enrichment analysis implied that two pairs of the genes shared numerous biological processes (BP) (Figures 5(a) and 5(b)), that is, regulation of lipid metabolic processes and lipid localization; cellular response to lipid associated with metabolism of lipid; regulation of MAP kinase activity and protein kinase activity associated with regulation of kinases; regulation of coagulation responded to wounding; negative regulation of cell proliferation and apoptotic signaling pathway relevant cell cycle; and so on. Nevertheless, referring to molecular functions (MF) (Figures 5(c) and 5(d)) and cellular components (CC) (Figures 5(e) and 5(f)), there were less shared ways than BP; for example, MF shared lipid and phosphatase binding, receptor ligand, growth factor, receptor ligand, growth factor activities, etc. And CC showed similar membrane raft, receptor complex, membrane region, and so forth. In addition, KEGG signaling pathways (Figure 6) explicating hypolipidemic effect were involved in processes including metabolism associated with AGE-RAGE signaling pathway in diabetic complications, adipocytokine signaling pathway, and insulin signaling pathway; kinase function associated with MAPK signaling, AMPK signaling, and PI3K-Akt signaling pathways; cell cycle related to apoptosis, and so on communally in two groups of targets; interestingly, we found that more pathways related to shared lists, potential targets list and fewer ones belonging to candidate targets one; more details about shared and unique enrichment analysis information were listed in Supplementary Tables 5 and 6. Though the enrichment analysis, we show that the targets of WWQZS against hyperlipidemia were associated with lipid metabolism processes and phosphatase binding function as well as kinase-related pathways.

### 3.5. Effect of WWQZS on Body Mass of Hamsters

**Figure 7:**
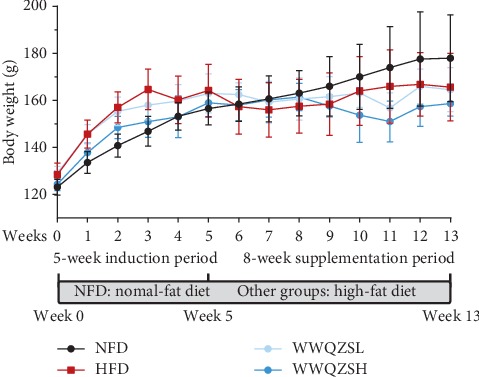
The effect of WWQZS on the weight mass in hamsters. In the induction phase, hamsters were separated into the NFD group (*n* = 6) and HFD group (*n* = 18). After 5 weeks, the 18 hamsters were randomly assigned to three groups (six hamsters/one group) by administration of 7.8 or 3.12 g/kg concentration of WWQZS.

During the 5-week induction period, the rates of hamsters' body mass fed on HFD were faster than the ones fed on NFD. After induction time, the weight of hamsters fed on NFD sustainably increased but the HFD groups began to fluctuate; interestingly, even the weight had reduced. In general, the weight of WWQZH group descended more than that of the HFD group (Figure 7).

### 3.6. Effect of WWQZS on TC, TG, LDL-C, and HDL-C in Serum

**Figure 8:**
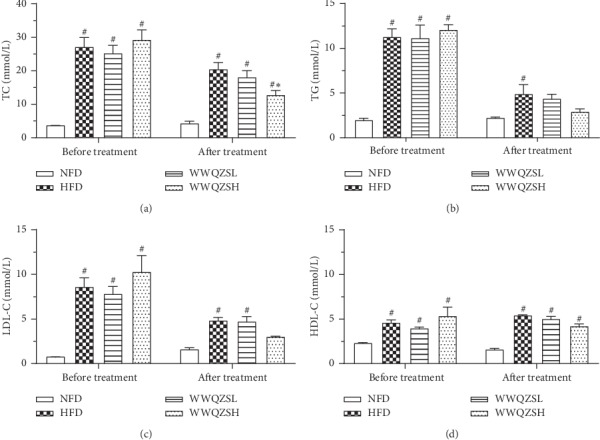
The effect of WWQZS on serum lipid in hamster (a–d). Respectively, the serum TC, TG, LDL-C, and HDL-C levels are presented before and after treatment in all groups. Values are represented as mean ± SD. ^#^*P* < 0.05 vs. NFD and ^*∗*^*p* < 0.05 vs. HFD.

To investigate whether WWQZS has an influence on the blood lipid level of HFD-induced hyperlipidemia hamsters, we measured serum TC, TG, LDL-C, and HDL-C both before and after administration of WWQZS.  Figure 8 shows that compared with NFD before treatment, the serum TG, TC, LDL-C, and HDL-C were all elevated significantly different in HFD hamsters (*P* < 0.05, *n* = 6) (Figures 8(a)–8(d)). After administration of the 7.8 or 3.12 g/kg concentration of WWQZS for 8 weeks, the serum TC and TG in HFD group significantly rose (*P* < 0.05, *n* = 6) (Figures 8(a) and 8(b)) compared with the NFD, but compared to HFD, the TC, TG, LDL-C, and HDL-C in the WWQZH group were significantly decreased (*P* < 0.05, *n* = 6) (Figures 8(a)–8(d)); meanwhile, the TG and LDL-C of WWQZSL and WWQZSH groups were reduced but not significantly (Figures 8(b) and 8(c)). However, treatment with WWQZS had no significant influence on HDL-C. These results implied that WWQZS could reduce the serum TC, TG, and LDL-C to influence the serum lipid.

### 3.7. Histological Analysis of WWQZS on Liver

**Figure 9:**
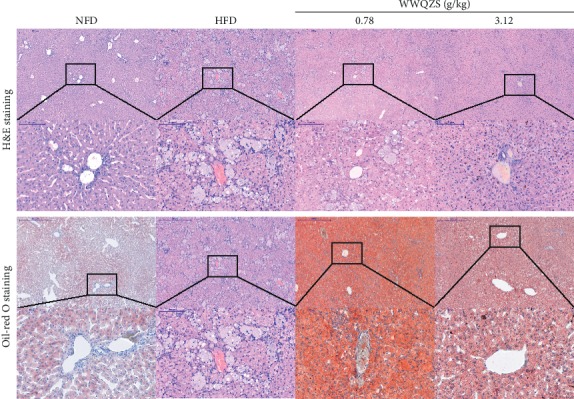
The effect of WWQZS on impaired liver induced by lipid accumulation in hamsters. Tissue sections of a representative liver section from each group (HFD; NFD; WWQZL; and WWQZH) were stained with H&E (a–d,×50, bar = 500 *μ*m; a–d; ×200, bar = 100 *μ*m) and oil-red O (E–H × 50, bar = 500 *μ*m; e–h,×200, bar = 100 *μ*m).

Liver sections in NFD group showed that the structures of tissues were normal with polygonal edges and clear cell borders, and the *nucleus* stayed round and clear (Figure 9, A, a). In contrast, the HFD group revealed visible histological changes including cell edema, focal degeneration, and necrosis (Figure 9 B, b). Likewise, there was degeneration in WWQZSL group but much better than the ones in HFD (Figure 9 C, c). However, the tissue in WWQZSH nearly returned to normal and cell edema nearly was found with distinct and clear borders (Figure 9 D, d). Oil-red O staining revealed the presence of lipid accumulation in both HFD and WWQZSL groups (Figure 9 E–G, e-g) but hepatic lipid accumulation in WWQZSH group was less than one in HFD (Figure 9 H, h).

### 3.8. Effect of WWQZS on AST and ALT in Serum

**Figure 10:**
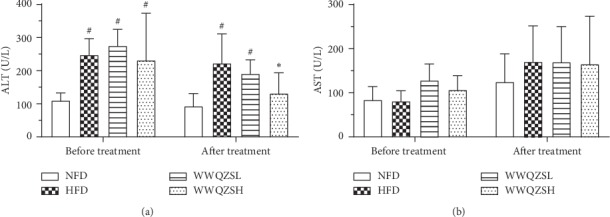
The effect of WWQZS on serum ALT and AST in hamster (a-b). Respectively, the serum ALT and AST level are presented before and after treatment in all groups. Values are represented as mean ± SD. ^#^*P* < 0.05 vs. NFD and ^*∗*^*p* < 0.05 vs. HFD.

We measured serum AST and ALT both before and after administration of *WWQZS* to confirm the liver function.  Figure 10 shows that compared with NFD before treatment, the serum ALT (Figure 10(a)) increased significantly in HFD hamsters (*P* < 0.05, *n* = 6) but had no influence on AST (Figure 10(b)). After administration of WWQZS for 8 weeks, the serum AST in HFD and WWQZSL groups significantly rose (*P* < 0.05, *n* = 6) (Figure 10(a)) compared with the NFD, but compared to HFD, it was significantly decreased in the WWQZH group (*P* < 0.05, *n* = 6) and decreased in WWQZSL group (Figure 10(a)); meanwhile, it still had no influence on AST (Figure 10(b)). These results implied that WWQZS could reduce the serum AST to influence the liver function.

### 3.9. The Underlying Mechanism of WWQZS for Lowering Cholesterol

**Figure 11:**
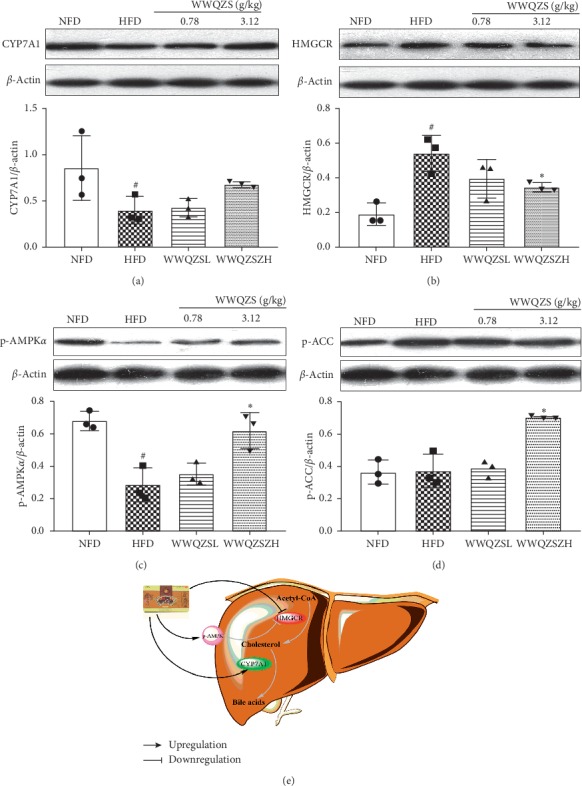
The underlying mechanism of WWQZS for lowering cholesterol. The expression of cholesterol catabolism and synthesis of vital proteins (CYP7A1, HMGCR) as well as upstream p-AMPKα and p-ACC were measured by Western blotting. Quantification of protein level was normalized to *β*-actin using densitometry (a–d), Values are represented as mean ± SD. ^#^*P* < 0.05 vs. NFD and ^*∗*^*p* < 0.05 vs. HFD. The figure illustrated that WWQZS upregulated CYP7A1 and p-AMPKα and downregulated HMGCR to decrease cholesterol based on this study (e).

Due to the significant effect of WWQZS for lowering cholesterol in pharmacology experiments above, the protein expression of *HMGCR*, CYP7A1, p-AMPK*α*, and p-ACC for cholesterol metabolism was evaluated in livers of WWQZS-treated hyperlipidemia hamsters by Western blotting. Compared with NFD group, WWQZS promoted the expression of CYP7A1 in two groups of WWQZS compared with HFD group; further, WWQZS attenuated the HMGCR protein expression significantly (*P* < 0.05) in WWQZSH group compared with HFD group (Figures 11(a) and 11(b)). The expression of both p-AMPKα and p-ACC increased significantly (*P* < 0.05) compared to HFD group, which showed that AMPK*α* was active. The underlying mechanism of WWQZS for lowering cholesterol was shown in Figure 11(e).

## 4. Discussion and Conclusion

Ethnomedicines, as a part of the traditional medicine system in China, have their unique traditional theories as guide for rational administration [18]. Research principles and methods for indigenous medicines should stay different but equivalently same to its policies [19]. For this reason, a strategy was proposed including effective bioactive constituents, clear mechanism, and theoretical, empirical characteristics of ethnomedicine [20]. Moreover, application of bioinformatics and systems biology served as new concepts figuring out the scientific basis and the systematic features of TCM formula, namely, network pharmacology [21, 22]; therefore, a method, network pharmacology, was brought into ethnomedicine research and was employed with the guide of characteristic theory basis.

Among multiple distinct systems of medical traditions in China, TMM is one of four ethnomedicine systems in China and plays a critical role in people's health in Inner Mongolia, China. TMM unique basic principles have assumed that human beings depend on three life-sustaining humors, *hii* (wind), *sar* (bile),and *badgan* (phlegm) [23] and seven elements, food essence, blood, muscle, fat, bone, and marrow essence fluid for lives with eliminating phlegm and producing essence; meanwhile, health is defined as a balance between three humors and hot/cold [24]. TMM implies food eaten by a human is not divided into phlegm and essence in the gastrointestinal tract and liver, called “Eliminate Phlegm and Produce Essence” (Qing Zhuo Sheng Hua in Chinese), causing enrichment of waste in the blood produced by the liver, which is similar to dyslipidemia in modern medicine. Due to the theory, Mongolian doctors use WWQZS to help the human body recover the function of “Eliminate Phlegm and Produce Essence” against hyperlipidemia, which differs in pesticide effect written in CH.P, which prompts a new medical use of WWQZS.

We established an HFD-induced hyperlipidemia hamster model, supplied with WWQZS, in order to confirm its anti-hyperlipidemic effect found on TMM theory; TC was significantly reduced by WWQZS and TG, LDL-C decreased as well; liver pathological section implied that WWQZS could relieve liver damage and lipid accumulation, which indicated that WWQZS has a better effect on serum TC and liver function. Through network pharmacology, we first used PPI to get potential targets and candidate targets to obtain the targets of WWQZS against hyperlipidemia; therefore, we overlapped 219 putative targets of 212 components and 349 known hyperlipidemia-related targets to get 43 potential targets and 45 candidate targets enrichment analysis, finding that BP shared numerous processes related to lipid metabolism, protein kinase activity in two groups of targets and MF and CC showed different kinds of lipid binding, phosphatase binding, receptor ligand activity activities, and cell components related to membrane raft, receptor complex, but shared less function and components in common. Furthermore, KEGG analysis showed lots of pathways related to metabolism and kinase activities. Interestingly, AMPK pathway, highly enriched, was closely related to cholesterol synthesis metabolism.

Thus, a further confirmation was to be carried out to detect the protein expression of p-AMPK, p-ACC, CYP7A1, and HMGCR by Western blot for the mechanism of lower cholesterol. As reported, phosphorylated AMPK attenuates the proteolytic processing of SREBP-2, inhibiting HMGCR expression to reduce lipid accumulation in the liver [25]. So, some studies showed some bioactive component and some Chinese medicines could downregulate CYP7A1 expression to decrease cholesterol [26, 27].

In this study, network pharmacology and validation experiments were carried out to reveal potential underlying mechanisms of WWQZS against HFD-induced hyperlipidemia. Numerous compounds of WWQZS could have a good effect against hyperlipidemia through multiple targets by lots of biological processes and pathways with different cell components; meanwhile, WWQZS may increase cholesterol catabolism by upregulating CYP7A1 and inhibit cholesterol synthesis by upregulating p-AMPK to suppress the expression of HMGCR in the liver to reduce serum lipid and protect liver invalidation hyperlipidemia hamster experiment.

## Figures and Tables

**Table 1:** Detailed information of WWQZS contents.

Medicinal materials	Chinese name	Plant origin	Content (g)
Granati Fructus	Shiliu	The dried ripe fruits of *Punica granatum* L.	400
Carthami Flos	Honghua	The dried ripe flowers of *Carthamus tinctorius* L.	200
Piperis Longi Fructus	Bibo	The dried ears of *Piper longum* L.	50
Amomi Fructus Rotundus	Doukou	The dried ripe fruits of *Amomum kravanh* pierre ex gagnep. or *Amomum compactum* soland ex maton)	50
Cinnamomi Cortex	Rougui	The dried ripe cortices of *Cinnamomum cassia* presl.)	50

## Data Availability

The data used to support the findings of this study are available from the corresponding author upon request.

## Supplementary Materials

Supplementary Materials Supplementary materials include the following. Supplementary Table 1: information of compounds from five herbal medicines in WWQZS collected from public data resources and Information of candidate compounds of five herbal medicines in WWQZS after screening. Supplementary Table 2: the details of targets from compounds in Shiliu, Honghua, Bibo, Doukou, and Rougui. Supplementary Table 3: known hyperlipidemia-related targets. WWQZS shared 43 potential targets with known anti-hyperlipidemia targets. Supplementary Table 4: the degrees and betweennesses of nodes in 45 candidate targets network. Supplementary Table 5: the BP, MF, and CC enrichment analyses for targets of WWQZS against hyperlipidemia. Supplementary Table 6: the KEGG pathways enrichment analysis for targets of WWQZS against hyperlipidemia.
